# One-year outcomes of thoracic endovascular stent graft repair for acute type B aortic penetrating ulcer combined with antiplatelet drugs

**DOI:** 10.3389/fcvm.2026.1676128

**Published:** 2026-05-26

**Authors:** Zhan-kui Du, Ya-song Wang, Ting-hao Zhao, Hou-lin He, Zhi-qiang Zhang, Xiao-zeng Wang

**Affiliations:** 1 Department of Cardiology, the Second Hospital Affiliated to Xi’an Medical University, Xi’an, Shaanxi, China; 2 State Key Laboratory of Frigid Zone Cardiovascular Disease, Cardiovascular Research Institute and Department of Cardiology, General Hospital of Northern Theater Command, Shenyang, Liaoning, China

**Keywords:** acute penetrating aortic ulcers, antiplatelet, coronary heart disease, prognosis, repair, thoracic endovascular

## Abstract

**Objective:**

The aim of this study was to investigate the 12—month clinical outcomes and 6—month morphological changes of acute penetrating aortic ulcers (PAUs) after thoracic endovascular aortic repair (TEVAR) combined with antiplatelet (AP) drugs.

**Methods:**

Patients who underwent TEVAR for acute PAUs at the General Hospital of Northern Theater Command from January 2012 to June 2024 were included. Demographics, clinical data, and imaging characteristics were retrospectively collected. The primary outcome was major adverse events, defined as a composite of death, endoleak, aortic rupture or re-dissection, re-intervention, stroke, acute myocardial infarction, and hemorrhage (BARC ≥ 2 grade).

**Results:**

Of the 195 patients, 59 were treated with AP drugs (AP group) and 136 without AP drugs (NAP group). There were no significant differences in preoperative demographic or imaging characteristics between the two groups. A total of 180 patients with acute PAUs underwent CTA reexamination within 6 months after TEVAR. At 6-month follow-up, the mean imaging parameters were as follows: aortic diameter at PAU, 32.44 ± 2.19 mm; PAU diameter, 1.83 ± 1.77 mm; PAU depth, 1.46 ± 0.96 mm; and intramural hematoma (IMH) thickness, 1.16 ± 1.03 mm. There were no significant differences in these aforementioned parameters between the AP and NAP groups. Compared with preoperative imaging parameters, neither group showed a significant difference in aortic diameter at PAUs; however, PAU diameter and depth were smaller in both the AP and NAP groups (all *P* < 0.001), and IMH thickness was also smaller in both groups (all *P* < 0.001). During 12-month follow-up, 31 patients (15.8%) experienced a primary outcome event. The cumulative incidence of major adverse events at 30 days and 12 months was higher in the AP group than in the NAP group (5.1% *vs*. 4.4% at 30 days; 18.6% *vs*. 14.7% at 12 months), but there were no statistically significant differences between the two groups. Cox regression analysis showed that maximum aortic diameter, PAU diameter ≥ 10.5 mm, and PAU depth ≥ 7.5 mm were associated with major adverse events.

**Conclusion:**

The present study indicated that AP therapy may be safe for patients with acute PAUs who underwent TEVAR. Maximum aortic diameter, PAU diameter ≥ 10.5 mm, and PAU depth ≥ 7.5 mm were associated with major adverse events.

## Introduction

Penetrating aortic ulcers (PAUs) are a subset of acute aortic syndrome (AAS), defined as the rupture of atherosclerotic lesions through the internal elastic lamina of the aortic wall with subsequent hematoma formation between the media and the adventitia (1). PAUs, a special type of AAS that is similar to aortic dissection (AD) and intramural hematoma (IMH) in terms of clinical manifestations, have unique pathological features and clinical outcomes distinct from other types of AAS (2). PAUs account for 2%–7% of AAS cases and may progress to AD, aneurysm formation, or aortic rupture (3).

Thoracic endovascular aortic repair (TEVAR) offers an effective approach to treating PAUs, with recognized efficacy in reducing mortality and morbidity (4). In clinical practice, patients with PAUs often have comorbid coronary heart disease (CHD) (5), and these patients may require percutaneous coronary intervention (PCI) after TEVAR or necessitate antiplatelet (AP) therapy (6).

In accordance with the 2014 consensus guidelines for the management of descending thoracic PAUs, AP therapy is recommended for the chronic phase (7). However, there are only a few case reports on acute PAUs treated with AP therapy, as AP drugs may lead to complications such as aortic wall rupture and bleeding (8).

The aim of this study was to retrospectively investigate the 12-month clinical outcomes and 6-month morphological changes in acute PAUs after TEVAR combined with AP drugs.

## Methods

### Patient selection

**Figure 1:**
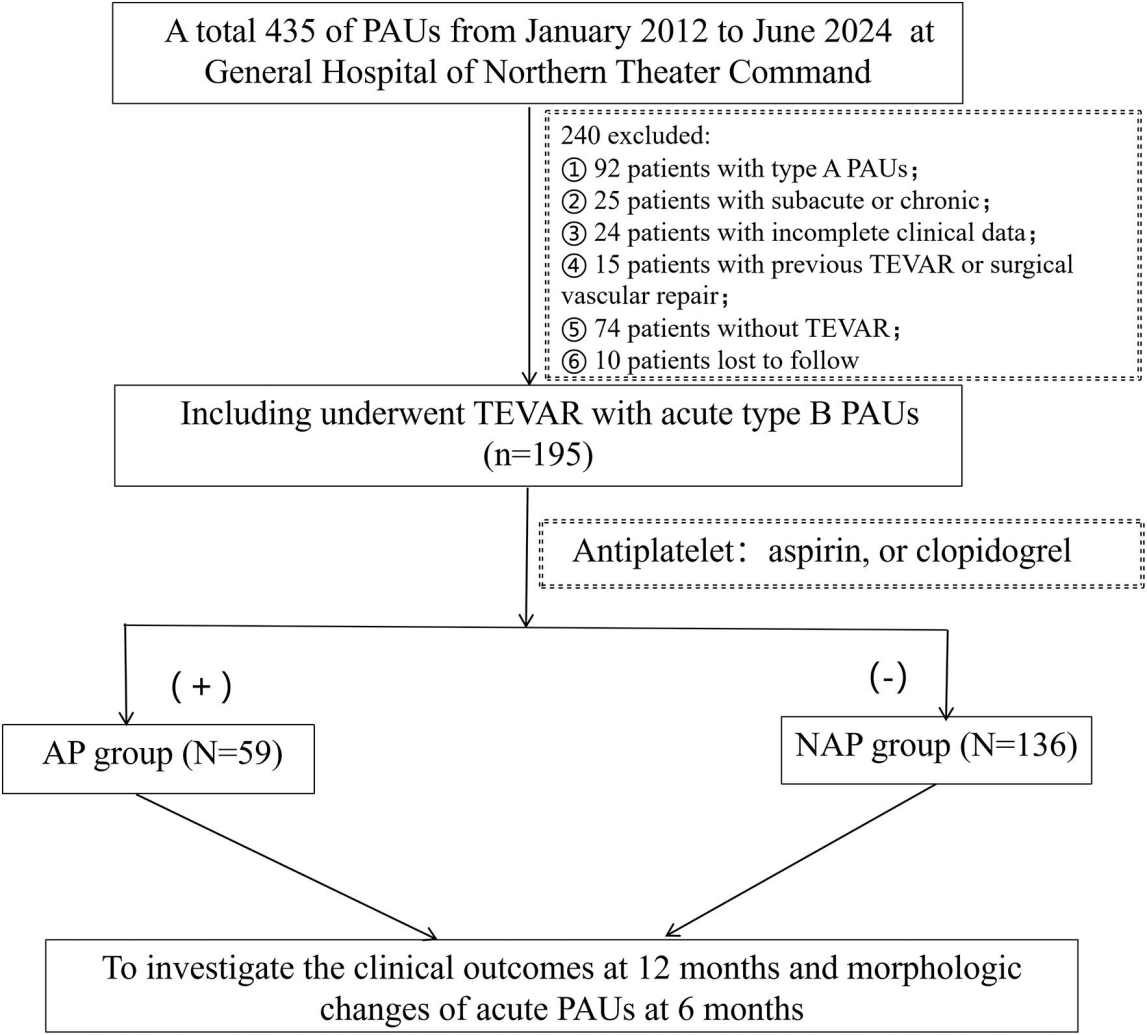
The flow chart. PAU, penetrating aortic ulcer.

This retrospective non-randomized study included consecutive patients with acute PAUs from January 2012 to June 2024 at the Department of Cardiology, General Hospital of Northern Theater Command. The diagnosis was confirmed by computed tomography angiogram (CTA) in all cases. PAUs were defined as focal ulcerations of the aorta with penetration of the internal elastic lamina into the aortic media, in the absence of aortic dissection (1). All patients were either acutely symptomatic or had an aortic ulcer in a state of AAS. The main exclusion criteria were: type A PAUs; subacute or chronic PAUs; incomplete clinical data; previous TEVAR or surgical vascular repair; failure to undergo TEVAR; and loss to follow-up. The flow chart is shown in Figure 1.

### Deﬁnitions and clinical management

Demographics, clinical characteristics, and preoperative and postoperative variables were collected. The acute phase was defined as less than 2 weeks from symptom onset. All operators were senior interventional cardiologists from the same department, each with at least five years of experience in TEVAR. This ensured a consistent level of technical proficiency and device familiarity throughout the study period. Additionally, all procedures were conducted following standardized institutional protocols to minimize operator-related variability.

All patients were managed according to a standardized clinical protocol throughout the study period. After confirmation of diagnosis, all patients were admitted to the intensive care unit for strict monitoring, control of pain and blood pressure, and underwent strict imaging follow-up with repeated aortic CTA at 1 month and 6 months from initial presentation. The timing of CTA was adjusted only if symptoms persisted or recurred.

AAS were considered complicated if they presented with rupture, impending rupture, or malperfusion. Endovascular treatment was performed for complicated cases, as suggested by current guidelines (9).

AAS were considered at high risk for complications if they had persistent pain or hypertension despite optimal medical therapy, progression to overt aortic dissection, an increase in aortic diameter greater than 5 mm, an increase in PAU depth or IMH thickness greater than 5 mm, or a combination of these factors; high-risk patients were considered for treatment. Refractory pain was defined as pain persisting for more than 5 days despite optimal medical therapy, after excluding other possible sources of similar pain (including cardiac causes, pulmonary embolism, pulmonary conditions, other causes of abdominal/thoracic pain, etc.) (10).

Follow-up consisted of CTA at 1 month and 6 months, and assessment of clinical outcomes at 1 month and 12 months after discharge.

### Image analysis

Baseline morphological characteristics and anatomical changes were measured from admission CTA. The maximum aortic diameter was measured as the outer-to-outer wall distance perpendicular to the centerline. PAU location, depth, and width were evaluated as previously reported (11). In cases of multiple PAUs, the major PAUs were considered for morphologic assessment. IMH thickness (maximum distance from intima to adventitia perpendicular to the centerline) and longitudinal extension were assessed. Proximal and distal extension of IMH were evaluated according to current reporting standards using the classification of aortic zones; this allows for assessment of disease extension relative to the level of aortic involvement, independent of anthropometric characteristics. To ensure reproducibility, images were reviewed by two independent physicians.

### Primary endpoint

The primary endpoint was major adverse events, including all-cause mortality, aortic-related adverse events (endoleak, aortic rupture or re-dissection, revascularization, limb ischemia, organ failure involving the kidneys and gastrointestinal tract, and stroke), acute myocardial infarction, and hemorrhage (BARC ≥ 2), assessed at 1 month and 12 months.

### Statistical analysis

This study reported categorical variables as numbers and percentages, and continuous variables as mean ± standard deviation or median with range. For continuous variables, comparisons were performed using the Student's *t*-test or Mann–Whitney *U*-test, as appropriate. For categorical variables, the Pearson's chi-square test or Fisher's exact test was used. Time-dependent outcomes were analyzed using Kaplan–Meier estimates. Cox proportional hazards models were employed to assess the impact of baseline characteristics on major adverse events. Statistical analyses were performed using SPSS software (version 22.0; IBM Corp., Armonk, NY, USA). A two-tailed *P*-value < 0.05 was considered statistically significant.

## Results

### Baseline characteristics

A total of 195 consecutive patients who underwent TEVAR for acute PAUs were included in our center. These patients were divided into the AP group (treated with AP drugs, *n* = 59) and the NAP group (not treated with AP drugs, *n* = 136).

Among the 195 patients, the mean age was 62.99 ± 7.05 years. Of these, 134 (68.7%) were male and 151 (77.4%) had hypertension. Most patients (91.8%) presented with persistent pain at initial presentation; however, 17 (8.7%) had abdominal pain. There were no significant differences in demographic characteristics between the AP and NAP groups. Similarly, no significant differences were observed in clinical presentation between the two groups (Table 1).

**Table 1:** Baseline clinical data.

Items	All (*n* = 195)	AP (*n* = 59)	NAP (*n* = 136)	*P*
Demographic characteristics				
Age (years)	62.99 ± 7.05	62.49 ± 6.61	63.21 ± 7.25	0.518
>65 years, *n* (%)	82 (42.1)	22 (37.3)	60 (44.1)	0.431
Male, *n* (%)	134 (68.7)	39 (66.1)	95 (69.9)	0.617
Smoking, *n* (%)	121 (62.1)	37 (62.7)	84 (61.8)	1.000
Drinking, *n* (%)	88 (45.1)	28 (47.5)	60 (44.1)	0.754
Hypertension, *n* (%)	151 (77.4)	48 (81.4)	103 (75.7)	0.458
Diabetes, *n* (%)	13 (6.7)	4 (6.8)	9 (6.6)	1.000
Clinical presentation				
Chest pain, *n* (%)	168 (86.2)	50 (84.7)	118 (86.8)	0.822
Back pain, *n* (%)	172 (88.2)	53 (89.8)	119 (87.5)	0.810
Abdominal pain, *n* (%)	17 (8.7)	6 (10.2)	11 (8.1)	0.594
Persistent pain, *n* (%)	179 (91.8)	52 (88.1)	127 (93.4)	0.220
Antiplatelet				
Aspirin, *n* (%)	47 (24.1)	47 (79.7)	-	-
Clopidogrel, *n* (%)	21 (10.8)	20 (35.6)	-	-
Dul-antiplatelet, *n* (%)	7 (3.6)	7 (11.9)	-	-

PAU, penetrating aortic ulcer; IMH, intramural hematoma.

### Preoperative and postoperative aortic morphology

At admission, the mean maximum aortic diameter was 42.70 ± 6.68 mm, the mean aortic diameter at PAUs was 30.94 ± 2.45 mm, and the mean PAU diameter and depth were 11.69 ± 3.36 mm and 9.55 ± 2.86 mm, respectively. The mean number of PAUs was 1.15 ± 0.51. There were no significant differences in these parameters between the two groups. Most ulcers (61.5%) were located in zone 3, with no significant difference between the two groups. Similar to PAUs, the mean IMH thickness was 12.72 ± 4.07 mm, and there were also no significant differences between the two groups. The proportions of IMH extension in zone 3, zone 4, and zone 5 were 45.1%, 34.4%, and 20.5% of all patients, respectively, with no significant differences between the two groups (Table 2).

**Table 2:** Imaging characteristics in preoperative.

Items	All (*n* = 195)	AP (*n* = 59)	NAP (*n* = 136)	*P*
Aortic maximum diameter, mm	42.70 ± 6.68	43.74 ± 6.82	42.25 ± 6.60	0.154
Aortic diameter at PAU, mm	30.94 ± 2.45	31.15 ± 2.67	30.85 ± 2.35	0.435
Mean number of PAU, *n* (%)	1.15 ± 0.51	1.18 ± 0.54	1.14 ± 0.48	0.620
PAU diameter, mm	11.69 ± 3.36	11.91 ± 3.56	11.60 ± 3.28	0.553
PAU depth, mm	9.55 ± 2.86	9.81 ± 2.53	9.44 ± 2.99	0.415
PAU aortic location, *n* (%)				0.749
Zone 3, *n* (%)	120 (61.5)	35 (59.3)	85 (62.5)	
Zone 4, *n* (%)	75 (38.5)	24 (40.7)	51 (37.5)	
IMH thickness, mm	12.72 ± 4.07	12.84 ± 4.18	12.66 ± 4.03	0.780
IMH extension, *n* (%)				1.000
Zone 3, *n* (%)	88 (45.1)	27 (45.8)	61 (44.9)	
Zone 4, *n* (%)	67 (34.4)	20 (33.9)	47 (34.6)	
Zone 5, *n* (%)	40 (20.5)	12 (20.3)	28 (20.6)	
Coronary stenosis > 50%, *n* (%)	59 (30.2)	59 (100.0)	–	–
Coronary stenosis > 70%, *n* (%)	25 (12.8)	25 (42.3)	–	–

**Figure 2:**
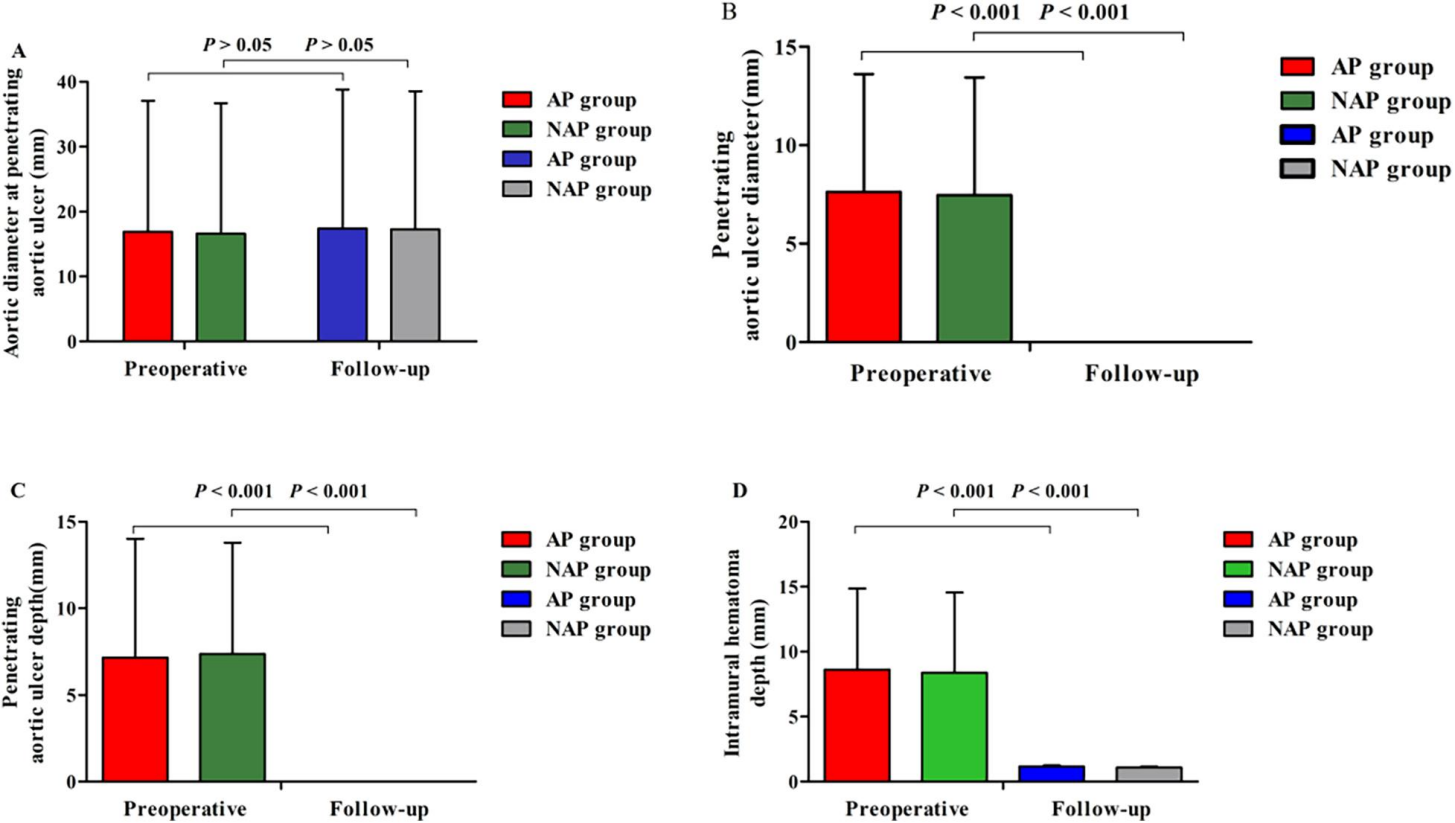
Aortic morphological changes at 6 months follow-up. **(A)** Changes of aortic diameter at penetrating aortic ulcer in preoperative and postoperative. **(B)** Changes of penetrating aortic ulcer diameter in preoperative and postoperative. **(C)** Changes of penetrating aortic ulcer depth in preoperative and postoperative. **(D)** Changes of intramural aortic hematoma depth in preoperative and postoperative.

A total of 180 patients with acute PAUs underwent CTA reexamination within 6 months after TEVAR. At 6-month follow-up, the mean imaging parameters were as follows: aortic diameter at PAU, 32.44 ± 2.19 mm; PAU diameter, 1.83 ± 1.77 mm; PAU depth, 1.46 ± 0.96 mm; and IMH thickness, 1.16 ± 1.03 mm. There were no significant differences in these aforementioned parameters between the AP and NAP groups. Compared with preoperative imaging parameters: there were no significant differences in aortic diameter at PAUs between the two groups (AP group: 31.17 ± 2.67 mm *vs*. 32.55 ± 2.25 mm; NAP group: 30.79 ± 2.38 mm *vs*. 32.33 ± 2.22 mm; all *P* > 0.05); PAU diameter and depth were smaller in both the AP and NAP groups (AP group: PAU diameter, 11.85 ± 3.39 mm *vs*. 1.82 ± 1.13 mm; PAU depth, 12.01 ± 2.31 mm *vs*. 1.45 ± 0.99 mm; NAP group: PAU diameter, 11.69 ± 3.26 mm vs. 1.79 ± 1.17 mm; PAU depth, 11.91 ± 2.83 mm *vs*. 1.46 ± 0.94 mm; all *P* < 0.001); IMH thickness was also smaller in both groups (AP group: 13.03 ± 4.19 mm *vs*. 1.23 ± 1.07 mm; NAP group: 12.75 ± 4.02 mm *vs*. 1.13 ± 1.03 mm; all *P* < 0.001) (Figures 2A–D).

### Procedural details and clinical outcomes

The technical success rate was 98.4% (192/195), as 3 patients developed type Ia endoleaks and were considered unsuccessful cases. Given the limited extent of these endoleaks, no additional interventions were performed. A total of 195 stent grafts were implanted. Procedural details and clinical outcomes are presented in Table 3.

**Table 3:** Procedural details and clinical outcomes.

Items	All	AP	NAP	*P*
**Procedural details**	***n* = 195**	***n* = 59**	***n* = 136**	
Interval (onset to TEVAR), days	18.16 ± 7.42	18.78 ± 8.53	18.53 ± 6.92	0.829
Procedure time, min,	88.85 ± 22.30	91.97 ± 21.61	87.49 ± 22.53	0.199
Endoleak, *n* (%)	3 (1.5)	1 (1.7)	2 (1.5)	0.907
Diameter of stent, cm	33.27 ± 3.04	33.12 ± 3.13	33.33 ± 3.01	0.656
Length of stent, cm	132.67 ± 29.19	131.51 ± 27.88	133.18 ± 29.82	0.715
LSA coverage, *n* (%)	46 (23.6)	15 (25.4)	31 (22.8)	0.716
**30-day outcome**	***n* = 195**	***n* = 59**	***n* = 136**	
**Major adverse events, *n* (%)**	15 (7.7)	5 (8.5)	10 (7.4)	0.787
Mortality, *n* (%)	1 (0.5)	0	1 (0.7)	0.509
Aortic-related adverse events, *n* (%)	10 (5.1)	3 (5.1)	7 (5.1)	0.986
Aortic-related deaths, *n* (%)	1 (0.5)	0	1 (0.7)	0.509
Endoleak, *n* (%)	3 (1.5)	1 (1.7)	2 (1.5)	0.907
Rupture or re-dissection, *n* (%)	2 (1.0)	0	2 (1.5)	0.349
Revascularization, *n* (%)	1 (0.5)	0	1 (0.7)	0.509
Ischemia of the limb, *n* (%)	4 (2.1)	1 (1.7)	3 (2.2)	0.817
Organ failure (renal and intestinal tract), *n* (%)	3 (1.5)	1 (1.7)	2 (1.5)	0.907
Stoke, *n* (%)	2 (1.0)	1 (1.7)	1 (0.7)	0.541
Acute myocardial infarction, *n* (%)	3 (1.5)	1 (1.7)	2 (1.5)	0.907
BARC ≥ 2 grade, *n* (%)	2 (1.0)	1 (1.7)	1 (0.7)	0.541
**12-month outcome**	***n* = 194**	***n* = 59**	***n* = 135**	
**Major adverse events, *n* (%)**	24 (12.4)	9 (15.5)	15 (11.1)	0.479
Mortality, *n* (%)	2 (1.0)	1 (1.7)	1 (0.7)	0.545
Aortic-related adverse events, *n* (%)	17 (8.8)	5 (8.5)	12 (8.9)	1.000
Aortic-related deaths, *n* (%)	1 (0.5)	0	1 (0.7)	0.507
Endoleak, *n* (%)	4 (2.1)	1 (1.7)	3 (2.2)	0.812
Rupture or redissection, *n* (%)	4 (2.1)	2 (3.4)	2 (1.5)	0.390
Revascularization, *n* (%)	3 (1.5)	1 (1.7)	2 (1.5)	0.912
Ischemia of the limb, *n* (%)	1 (0.5)	0	1 (0.7)	0.507
Organ failure (renal and intestinal tract), *n* (%)	4 (2.1)	1 (1.7)	3 (2.2)	0.812
Stroke, *n* (%)	3 (1.5)	1 (1.7)	2 (1.5)	0.912
Acute myocardial infarction, *n* (%)	4 (2.1)	2 (3.4)	2 (1.5)	0.390
BARC ≥ 2 grade, *n* (%)	3 (1.5)	2 (3.4)	1 (0.7)	0.169

TEVAR, thoracic endovascular aortic repair; LSA, left subclavian artery.

The incidence of 30-day adverse events was 4.6% (9/195), with the AP group showing a higher incidence than the NAP group (5.1% vs. 4.4%, respectively); however, the difference was not significant. The 30-day mortality rate was 0.5%, with no significant difference between the two groups (0% *vs*. 0.7%): 1 patient in the NAP group died from aortic rupture following re-dissection; 2 patients experienced rupture or re-dissection (with revascularization performed in 1); 2 patients had cerebral stroke; and 3 patients had acute myocardial infarction. Two patients had BARC ≥ 2 grade hemorrhage: 1 in the AP group and 1 in the NAP group (Table 3).

The incidence of 12-month adverse events was 11.3% (22/194), with a higher rate in the AP group than in the NAP group, though the difference was not significant (13.6% *vs*. 10.4% in the AP and NAP groups, respectively). Of all patients, 1 in the AP group died of aortic rupture and multiple organ dysfunction syndrome at 4 months after TEVAR, and 1 in the NAP group died of acute cerebral hemorrhage at 7 months (Table 3).

**Figure 3:**
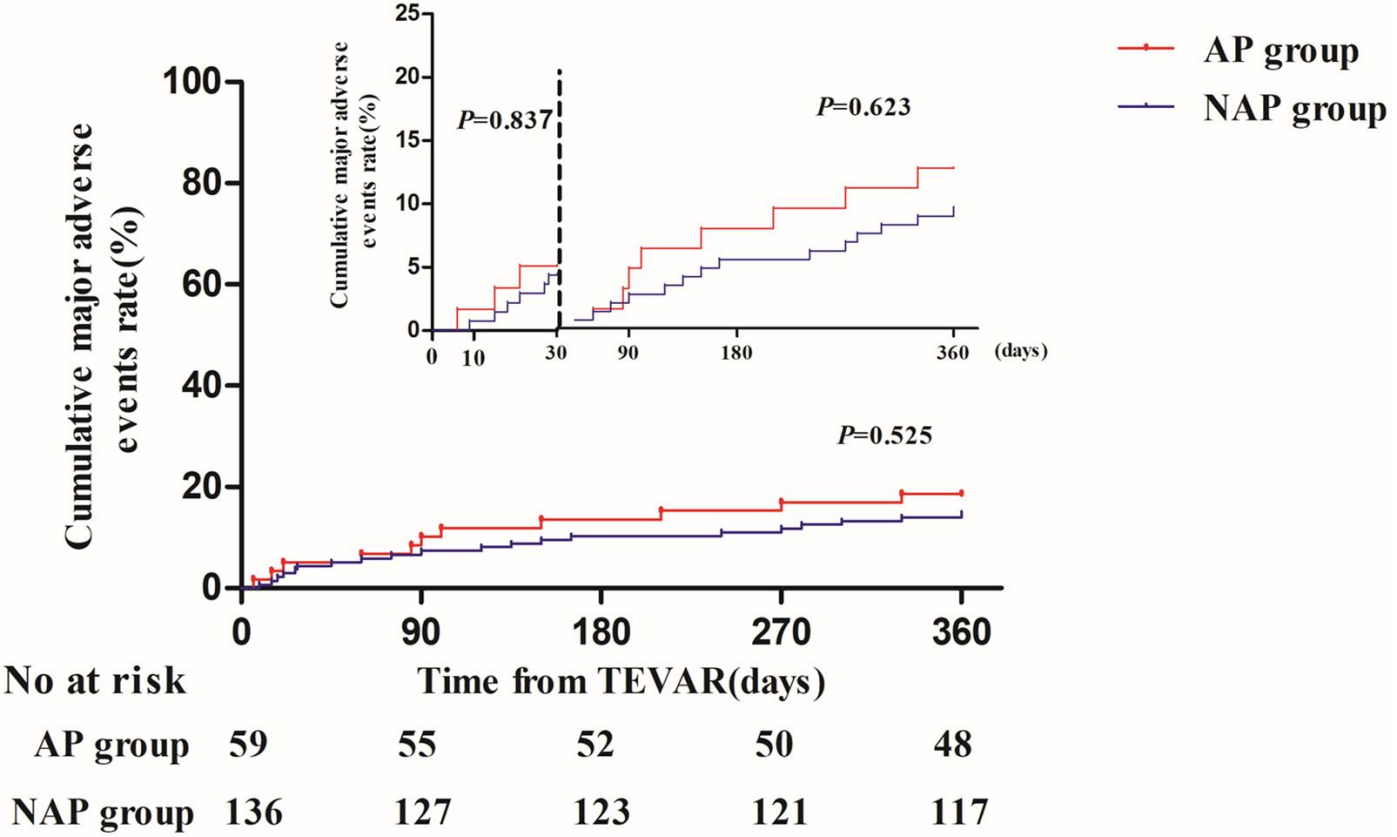
Survival curve of the clinical outcomes of all patients during follow-up.

The cumulative incidence of major adverse events at 30 days and 12 months was 4.6% and 15.9%, respectively, with 5.1% and 18.6% in the AP group and 4.4% and 14.7% in the NAP group, respectively. Log-rank tests showed no significant differences between the two groups (Figure 3). The cumulative incidence of mortality at the 12-month follow-up was 1.5% (1.7% vs. 1.4% for the AP group and the NAP group, respectively), and there were also no significant differences between the two groups.

### Predictive value of the PAUs diameter and depth for the prognosis

**Figure 4:**
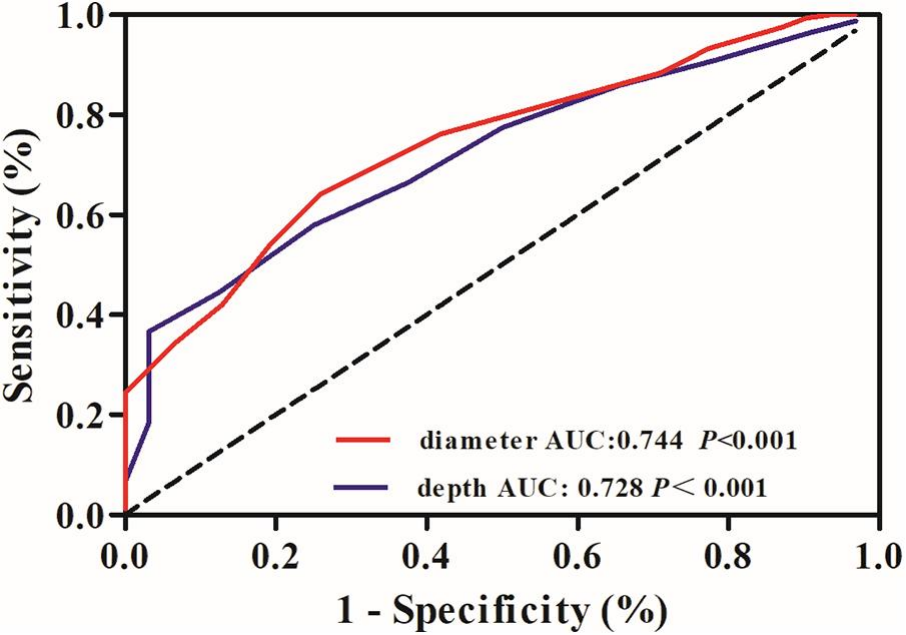
Receiver operating characteristic curve analysis for prediction of penetrating aortic ulcer based on the ulcer diameter and depth. AUC indicates area under the curve.

Receiver operating characteristic curve analysis demonstrated that PAU diameter and depth could distinguish patients who met the primary end point from those who did not. The optimal cutoff values for PAU diameter and depth were 10.5 mm and 7.5 mm, with areas under the curve of 0.744 and 0.728, respectively (Figure 4).

### Predictors of major adverse events: cox regression analysis

In the univariate analysis for major adverse events, variables with statistical significance (*P* < 0.05) included age >65 years, maximum aortic diameter, PAU diameter ≥ 10.5 mm, PAU depth ≥ 7.5 mm, and intramural hematoma (IMH) thickness. Additionally, antiplatelet (AP) drug use was included as a candidate indicator in the multivariate Cox regression models for major adverse events.

To identify factors influencing the prognosis of PAUs during the 12-month follow-up, Cox regression analysis was performed. The multivariate analysis revealed that maximum aortic diameter, PAU diameter ≥ 10.5 mm, and PAU depth ≥ 7.5 mm were independently associated with major adverse events (Table 4).

**Table 4:** Predictors of major adverse events: cox regression analysis.

Parameter	Univariate analysis			Multivariate analysis		
	HR	95% CI	*P* value	HR	95% CI	*P* value
Age >65 years	2.652	1.270–5.536	0.009	1.979	0.915–4.279	0.083
Hypertension	0.828	0.371–1.852	0.646			
Antiplatelet	1.300	0.623–2.714	0.484	0.788	0.360–1.729	0.553
Aortic maximum diameter	1.150	1.078–1.227	<0.001	1.100	1.032–1.174	0.004
Aortic diameter at PAUs	0.913	0.796–1.048	0.195			
PAUs diameter ≥ 10.5 mm	1.274	1.141–1.423	<0.001	1.250	1.111–1.407	<0.001
PAUs depth ≥ 7.5 mm	1.264	1.124–1.423	<0.001	1.216	1.066–1.386	0.003
IMH thickness	1.075	1.017–1.137	0.011	0.955	0.831–1.097	0.054

PAU, penetrating aortic ulcer; IMH, intramural hematoma.

## Discussions

To our knowledge, the present study is the largest retrospective analysis evaluating acute PAUs after TEVAR combined with AP drugs in the Chinese population. Additionally, we assessed the clinical features, evolution of clinical outcomes, imaging findings, and multivariate predictors of acute PAUs. This study yielded several key findings. First, there were no differences between the AP and NAP groups in 6-month morphological changes or 12-month clinical outcomes, suggesting that AP therapy may be safe and effective for patients with PAUs after TEVAR. Second, maximum aortic diameter, ulcer diameter ≥10.5 mm, and ulcer depth ≥7.5 mm were associated with major adverse events; moreover, PAU diameter ≥ 10.5 mm and PAU depth ≥ 7.5 mm may serve as cutoff parameters for PAU intervention.

It is well established that PAUs and CHD share common risk factors, such as age, hypertension, and diabetes. In the present study, 59 patients (30.2%) had coronary stenosis >50%, and 25 patients (12.8%) had coronary stenosis >70%. The 2014 ESC/EACTS Guidelines on Myocardial Revascularization recommend AP therapy for PCI in CHD, including long-term oral aspirin (75–100 mg daily) plus clopidogrel (75 mg daily) for 12 months (12). However, there is no consensus regarding the feasibility and safety of AP therapy in patients with acute PAUs who underwent TEVAR. In this study, we evaluated the safety and necessity of AP therapy for PAUs treated with TEVAR in patients with comorbid CHD, using primary endpoints such as mortality, aortic-related adverse events, acute myocardial infarction, and hemorrhage. Our results showed no significant differences at 1-month and 12-month follow-up, indicating that oral AP drugs were safe for PAU patients who underwent TEVAR. It is important to emphasize that, theoretically, the higher incidence of major events in the AP patients could be driven not by AP therapy itself, but by the underlying burden of systemic atherosclerosis (i.e., concomitant coronary) that necessitated AP therapy in the first place. However, there was no significant difference between AP and NAP in our study. Thus, from a clinical perspective, even if some residual confounding persists, the finding that AP patients experienced more events remains relevant: it identifies a population that, regardless of the exact mechanism, requires closer monitoring or more aggressive risk factor modification. Whether the increased risk is due to the drug or the disease, heightened vigilance in this subgroup is warranted.

Our findings suggest that the overall prognosis of patients with PAUs depends on ulcer depth, width, and the risk of underlying aortic pathology. In this study, ulcer diameter ≥10.5 mm and ulcer depth ≥7.5 mm were independent risk factors for major adverse events within 12 months. The 2024 European Society of Cardiology (ESC) guidelines on aortic diseases recommend early intervention for asymptomatic PAUs with diameters ≥13–20 mm or depths ≥10 mm (9). Our previous study suggested that patients with ulcer diameter >12.5 mm or depth >9.5 mm have a higher risk of disease progression, warranting early intervention (13). The present study provides intervention criteria for the Chinese population, with cutoff values of 10.5 mm for ulcer diameter and 7.5 mm for ulcer depth. This discrepancy may be attributed to two factors: First, it may reflect differences in the baseline characteristics of the study population, such as racial or differences in body habitus. Second, and more importantly, our thresholds were statistically derived to optimize predictive performance in this specific dataset, aiming to enhance early warning sensitivity. While racial or anthropometric differences (e.g., average body mass index) between Asian and European populations may play a role, the primary driver for our chosen values was the statistical fit to our outcome data. We suggest that clinicians interpret our findings as indicating that even ulcers slightly smaller than the ESC thresholds may still carry a significant risk in our population, warranting close attention.

Thoracic PAUs often follow a malignant course, making timely and effective intervention crucial. PAU formation is associated with atherosclerosis, which can eventually lead to AD in approximately 5% of patients (14, 15). Effective intervention is the optimal way to reduce PAU progression. TEVAR plays a potential preventive role by stabilizing these lesions and protecting the aorta from further damage (16). Regarding PAU interventions, several studies have demonstrated the safety of TEVAR across diverse presentations of thoracic PAUs and procedural urgency (elective vs. emergent), with consistently high technical success rates (92%) (17–19). However, the benefit of AP therapy in PAU patients with comorbid CHD is mainly attributed to its effects on arteriosclerosis (20). After ulcer occlusion by stenting, accelerated positive remodeling of the ulcer and intramural hematoma was observed, as confirmed by 6-month imaging follow-up.

## Limitations

The present study was a retrospective observational study with inherent limitations. First, the study population was relatively small, and the observational period was limited to 12 months because of its single-center design. As a result, the susceptibility to selection bias and measurement bias is increased. Thus, confirmation through prospective, multi-center registries with standardized long-term follow-up is required. Second, the extended study period from 2012 to 2024 introduces a potential temporal selection bias. Changes in diagnostic technologies, surgical techniques, or perioperative management over this period may have influenced patient selection criteria and outcomes. Third, the patients with more extensive AP often present with a higher prevalence of systemic atherosclerosis, which the potential for selection bias and residual confounding inherent to the retrospective design cannot be completely eliminated. Last, the cutoff values identified in this study require validation in larger cohorts to assess their generalizability and stability across different populations.

## Conclusion

The present study indicated that AP therapy may be safe for patients with acute PAUs underwent TEVAR in 12 months. Aortic maximum diameter, PAUs diameter ≥ 10.5 mm, and PAU depth ≥ 7.5 mm were associated with major adverse events. This requires validation in larger cohorts to assess their generalizability and stability.

## Data Availability

The raw data supporting the conclusions of this article will be made available by the authors, without undue reservation.
