# Supplementary material for: One-year outcomes of thoracic endovascular stent graft repair for acute type B aortic penetrating ulcer combined with antiplatelet drugs
Source: Front Cardiovasc Med. 2026 May 26;13:1676128. doi: 10.3389/fcvm.2026.1676128 (PMC13247544; doi:10.3389/fcvm.2026.1676128)
Supplement: Supplementary file 1 [file Table1.docx]

**Supplement table 1 .** Baseline clinical data .

| **Items** | **AP (n=59)** | **EAP (n=99)** | ***P*** |
| --- | --- | --- | --- |
| **Demographic characteristics** |  |  |  |
| Age (years) | 62.49±6.61 | 62.97±7.14 | 0.676 |
| > 65 years, *n* (%) | 22 (37.3) | 43 (43.4) | 0.505 |
| Male, *n* (%) | 39 (66.1) | 68 (68.7) | 0.861 |
| Smoking, *n* (%) | 37 (62.7) | 59 (59.6) | 0.738 |
| Drinking, *n* (%) | 28 (47.5) | 45 (45.5) | 0.870 |
| Hypertension, *n* (%) | 48 (81.4) | 76(76.8) | 0.553 |
| Diabetes, *n* (%) | 4(6.8) | 8 (8.1) | 1.000 |
| **Clinical presentation** |  |  |  |
| Chest pain, *n* (%) | 50 (84.7) | 87(87.9) | 0.631 |
| Back pain, *n* (%) | 53 (89.8) | 87(87.9) | 0.800 |
| Abdominal pain, n (%) | 6 (10.2) | 6 (6.1) | 0.366 |
| Persistent pain, *n* (%) | 52 (88.1) | 92 (92.9) | 0.387 |
| **Antiplatelet** |  |  |  |
| Aspirin, *n* (%) | 47 (79.7) | 66 (66.7) | 0.101 |
| Clopidogrel, *n* (%) | 20 (35.6) | 22(22.1) | 0.096 |
| Dul-antiplatelet, *n* (%) | 7 (11.9) | 5 (5.1) | 0.132 |

PAU, penetrating aortic ulcer; IMH, intramural hematoma; AP, antiplatelet; EAP, excluded antiplatelet.
